# Antimicrobial peptide LL-37 disrupts plasma membrane and calcium homeostasis in *Candida albicans* via the Rim101 pathway

**DOI:** 10.1128/spectrum.02551-23

**Published:** 2023-10-27

**Authors:** Sheng-Yuan Chen, Che-Kang Chang, Chung-Yu Lan

**Affiliations:** 1 Institute of Molecular and Cellular Biology, National Tsing Hua University, Hsinchu, Taiwan; 2 Department of Life Science, National Tsing Hua University, Hsinchu, Taiwan; 3 School of Medicine, National Tsing Hua University, Hsinchu, Taiwan; University of Guelph, Guelph, Ontario, Canada

**Keywords:** *Candida albicans*, LL-37, plasma membrane, mitochondria, vacuoles, calcium homeostasis, ROS, Rim101

## Abstract

Antimicrobial peptides play an important role in human innate defense against microbial pathogens. LL-37 is the only member of the human cathelicidin family of antimicrobial peptides. We previously showed that LL-37 targets the cell wall of *Candida albicans*, thereby reducing cell adhesion to plastic surfaces, oral epidermoid cells, and urinary bladders of mice. Moreover, LL-37 also induces cell wall and endoplasmic reticulum stress responses. Here, we found that LL-37 alters plasma membrane properties. In addition, LL-37 affected calcium homeostasis and induced the generation of reactive oxygen species associated with mitochondria and vacuoles. Finally, the Rim101 pathway was linked to the cellular response to LL-37. Together, these findings provide new insights into anti-*C*. *albicans* actions of LL-37 and highlight the complex cellular responses induced by LL-37.

**IMPORTANCE:**

*Candida albicans* is a major human fungal pathogen, and antimicrobial peptides are key components of innate immunity. Studying the interplay between *C. albicans* and human antimicrobial peptides would enhance a better understanding of pathogen-host interactions. Moreover, potential applications of antimicrobial peptides in antifungal therapy have aroused great interest. This work explores new mechanisms of LL-37 against *C. albicans* and reveals the complex connection among calcium homeostasis, oxidative stress, signaling, and possibly organelle interaction. Notably, these findings support the possible use of antimicrobial peptides to prevent and treat fungal infections.

## INTRODUCTION

*Candida albicans* is an important human fungal pathogen that can cause different infections, particularly in immunocompromised patients (1). Importantly, *C. albicans* is a leading cause of bloodstream infection with high mortality rates (2). Moreover, the emergence of antifungal drug resistance has also become a great threat to anti-*C*. *albicans* therapy (3). Therefore, the World Health Organization has recently listed *C. albicans* as one of the four critical priority fungal pathogens that require further research, drug development, and public health action (4).

Antimicrobial peptides (AMPs) are major components of human innate immunity and act as the first line of defense against a wide variety of microbial pathogens, including *C. albicans* (5). AMPs are generally short (<100 amino acids), positively charged, and amphipathic (6). Based on amino acid sequences and structural features, human AMPs are classified into different families that are exemplified by defensins, histatins, hepcidins, and cathelicidins (6, 7). Intriguingly, due to insignificant toxicity to the host and low resistance rates, AMPs are considered to be potential candidates for developing new antifungal agents (8, 9).

LL-37 is an alpha-helical peptide derived from proteolytic cleavage of the carboxyl-terminus of the only human cathelicidin, hCAP18 (10 – 12). LL-37 is found to be stored as a propeptide in specific neutrophil granules and expressed in various epithelial tissues (11, 13). LL-37 possesses antimicrobial but also antitumor and immunomodulatory activities (11, 12). To date, several studies have focused on investigating the effects of LL-37 on *C. albicans*. LL-37 is found to mainly remain associated with the *C. albicans* cell surface (14, 15). Our previous studies also revealed that LL-37 interacts with the cell surface through its binding to cell wall polysaccharides, especially mannans, and the major cell wall exoglucanase Xog1 (16 – 19). Moreover, LL-37 induces cell wall and endoplasmic reticulum (ER) stress responses, which are mediated by the transcription factor Sfp1 (20). Notably, treating with LL-37 leads to cell aggregation, cell wall remodeling, and β-glucan exposure in *C. albicans* (16, 17). Consequently, LL-37 reduces the adherence of *C. albicans* to plastic surfaces, oral epidermoid cells, and urinary bladders of mice (16).

The plasma membrane (PM) of *C. albicans* functions as a selectively permeable barrier that only allows certain substances to pass through. *C. albicans* has thus evolved to contain different PM-embedded transporters to uptake nutrients, organic acids, and ions (21). Moreover, the PM is a major target for various antifungal drugs and AMPs (22, 23). Nevertheless, many cell wall synthetic enzymes and cell wall glycosylphosphatidylinositol-anchor proteins reside at the PM (24). As aforementioned, LL-37 greatly impacts the cell wall of *C. albicans*. Because the PM is physically linked to the cell wall and affects each other (25), it is of interest to investigate whether or not LL-37 also targets the PM.

In this study, we examined the effects of LL-37 on *C. albicans* PM and the following consequences in cells with LL-37 treatment. We showed that LL-37 alters various PM properties, suggesting the disruption of the *C. albicans* PM. Moreover, LL-37 was found to affect calcium homeostasis and induce the production of reactive oxygen species (ROS), which are somehow associated with mitochondria and vacuoles. Finally, the Rim101 pathway is involved in the LL-37-mediated changes in calcium homeostasis and *C. albicans* killing. Together, this study provides further insights into the complex mechanisms of LL-37 against *C. albicans*.

## RESULTS

### LL-37 affects the PM properties in *C. albicans*

The PM plays many critical physiological roles and is the primary target of the antifungal drugs polyenes and azoles. PM potential (Δψ) is the difference in total charge between the outside and inside of the membrane (26). The inability to maintain Δψ is one of the indicators of PM damage. In addition, PM permeability is crucial for regulating the exchange of molecules and ions (25). Finally, lipid droplets are cytosolic organelles involved in lipid metabolism and storage, maintenance of membrane, prevention of lipotoxicity, and stress responses (27, 28). Interestingly, lipid droplet accumulation, activated by membrane-perturbing antifungal agents, is part of the membrane-associated stress responses in *C. albicans* (29).

**Fig 1:**
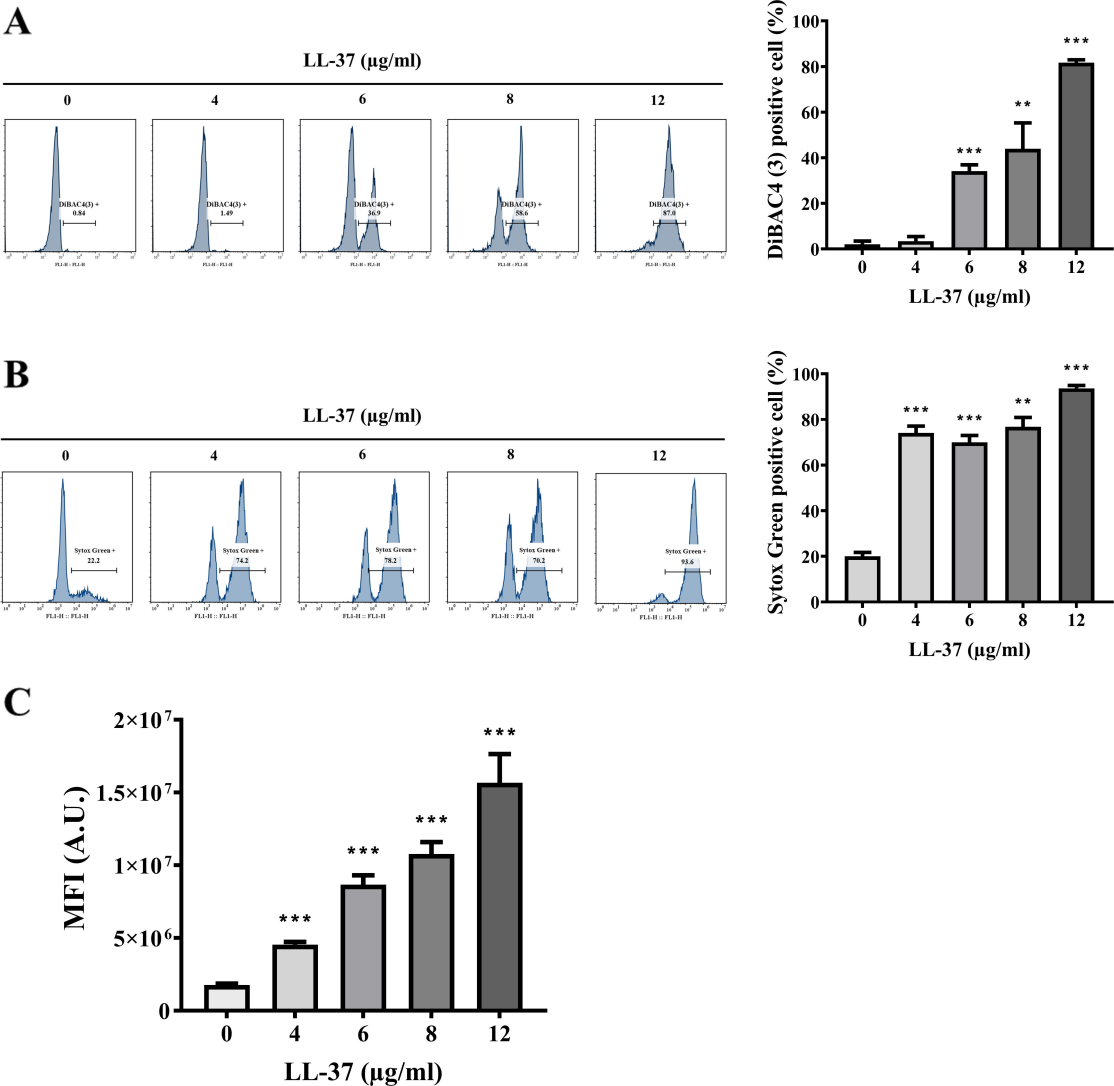
LL-37 affects the plasma membrane in *C. albicans*. The PM Δψ of cells was measured by (A) DiBAC4(3) staining, and the PM permeability was measured by (B) Sytox Green staining and analyzed using a flow cytometer. The percentages of DiBAC4(3)-positive and Sytox Green-positive cells were calculated. The results are presented as the mean ± standard deviation (SD) of three independent experiments. (C) Lipid droplet accumulation was measured by Nile red staining and analyzed using a flow cytometer. A.U.: arbitrary units. The results are presented as the mean ± SD of three independent experiments. **P < 0.01 and ***P < 0.001.

To determine whether LL-37 can act on the PM, Δψ was measured using DiBAC4(3) [bis-(1,3-dibutylbarbituric acid) trimethine oxonol] staining. DiBAC4(3) is a slow-response, membrane potential-sensitive fluorescence dye that only enters depolarized cells. As evaluated by flow cytometry histograms and the percentage of DiBAC4(3)-positive cells, an increase in the fluorescence intensity of DiBAC4(3) was detected with increasing concentrations of LL-37 (Fig. 1A), indicating that LL-37 induces membrane depolarization. Moreover, PM permeability was also determined using the Sytox Green stain. Sytox Green does not cross the intact PM but can easily penetrate compromised PM and exhibit strong green fluorescence upon binding nucleic acids (30). Indeed, the number of Sytox Green-positive cells was largely increased in *C. albicans* cells treated with LL-37 compared to the control without LL-37 treatment (Fig. 1B), suggesting membrane perturbation caused by LL-37. Finally, lipid droplet accumulation was also assessed further to determine the effects of LL-37 on the PM. Nile red is a lipophilic, polarity-sensitive, selective dye for intracellular lipid droplets (31). Nile red shows intense fluorescence in a lipid-rich environment, but the fluorescence quenching occurs in a more polar aqueous condition (32). In Fig. 1C, the mean fluorescence intensity (MFI) of Nile red was enhanced in cells treated with increasing concentrations of LL-37 compared to that without treatment. Together, these findings indicate that LL-37 causes changes in the PM and is likely to induce membrane-associated stress responses.

### LL-37 disrupts intracellular calcium equilibrium

Ions play a vital role in *C. albicans* and other living organisms, including membrane potential maintenance (33). For example, intracellular calcium concentrations are modulated in association with PM depolarization (34). In *C. albicans*, calcium is essential for the survival and pathogenicity of this pathogen (35). Moreover, the Cch1-Mid1 calcium channel, important for maintaining intracellular calcium concentration, is PM-localized (36). Intriguingly, various membrane-targeting AMPs can cause impairment of calcium homeostasis, leading to cell death (23).

**Fig 2:**
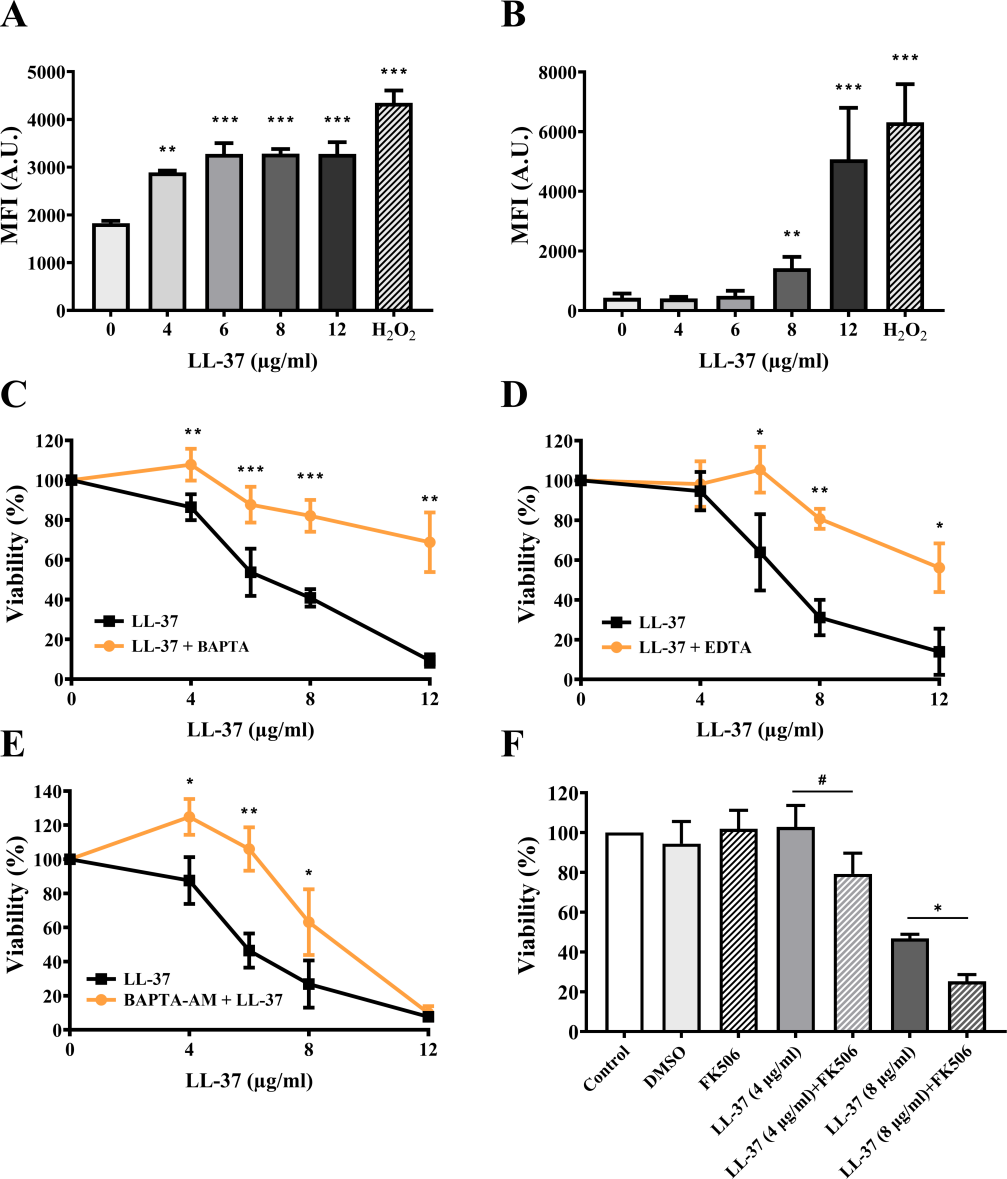
LL-37 disrupts intracellular calcium equilibrium. The cytosolic calcium levels were measured by (A) Flou-3 AM and (B) Fura-2 AM staining and flow cytometry. A.U.: arbitrary units. The correlation between the killing activity of LL-37 and cytosolic calcium was also determined. Cells were pretreated with extracellular calcium chelators (C) BAPTA, (D) EDTA, and an intracellular calcium chelator (E) BAPTA-AM, followed by treatment with different concentrations of LL-37 as indicated. Cell viability was calculated by counting the number of CFUs and expressed as percentage with respect to the control without LL-37 treatment. (F) To evaluate the combined effect of LL-37 and the calcineurin inhibitor FK506, cells were co-treated with LL-37 (4 µg/mL or 8 µg/mL) and FK506 [16 µg/mL; dissolved in dimethyl sulfoxide (DMSO)]. Cell viability was calculated by CFU counting and expressed as percentage. The results are expressed as mean ± SD of three independent experiments. All the results are expressed as mean ± SD of three independent experiments. *P < 0.05, **P < 0.01, ***P < 0.001, and #*P* = 0.07.

Because LL-37 affects distinct properties of the PM (Fig. 1A through C), we suspected the existence of a relationship between LL-37 and calcium homeostasis. To test this possibility, two membrane-permeant calcium-sensitive dyes, Fluo-3 AM and Fura-2 AM, were used independently to measure intracellular calcium in cells with LL-37 treatment. After entering cells, Fluo-3 AM and Fura-2 AM are cleaved by cellular esterases to Fluo-3 and Fura-2, respectively. Fluo-3 and Fura-2 bind to free intracellular calcium, increasing fluorescence intensity (37, 38). In Fig. 2A and B, the MFI of cells was increased due to the LL-37 treatment, compared to that of the untreated control. Moreover, to further validate the link between LL-37 and calcium homeostasis, the candidacidal activity of LL-37 was examined in cells pretreated with or without extracellular calcium chelators BAPTA and EDTA, and an intracellular calcium chelator BAPTA-AM. In Fig. 2C through E, cells were pretreated with the calcium chelating agents, washed, and treated with and without LL-37. The overall viability was much higher in cells treated with LL-37 than that without pretreatment. This result indicated that calcium chelation could affect *C. albicans* killing by LL-37. Finally, calcium/calcineurin signaling plays a crucial role in regulating calcium homeostasis in *C. albicans* (35). After combination treatment with LL-37 and the calcineurin inhibitor tacrolimus (FK506), *C. albicans* cells showed a lower survival rate than those treated with LL-37 alone (Fig. 2F). Together, our results prove that altered calcium homeostasis, either by calcium chelation or blocking the calcium/calcineurin pathway, correlates with LL-37-mediated *C. albicans* killing.

### LL-37 also induces mitochondrial calcium accumulation

Mitochondria are the primary site for energy production and are involved in various cellular activities, including stress responses, metabolism, and regulation of intracellular calcium homeostasis for cell signaling (39). Moreover, previous studies indicated that the candidacidal activity of various AMPs is related to elevated intracellular calcium, mitochondrial calcium influx, and mitochondrial dysfunction (40, 41).

**Fig 3:**
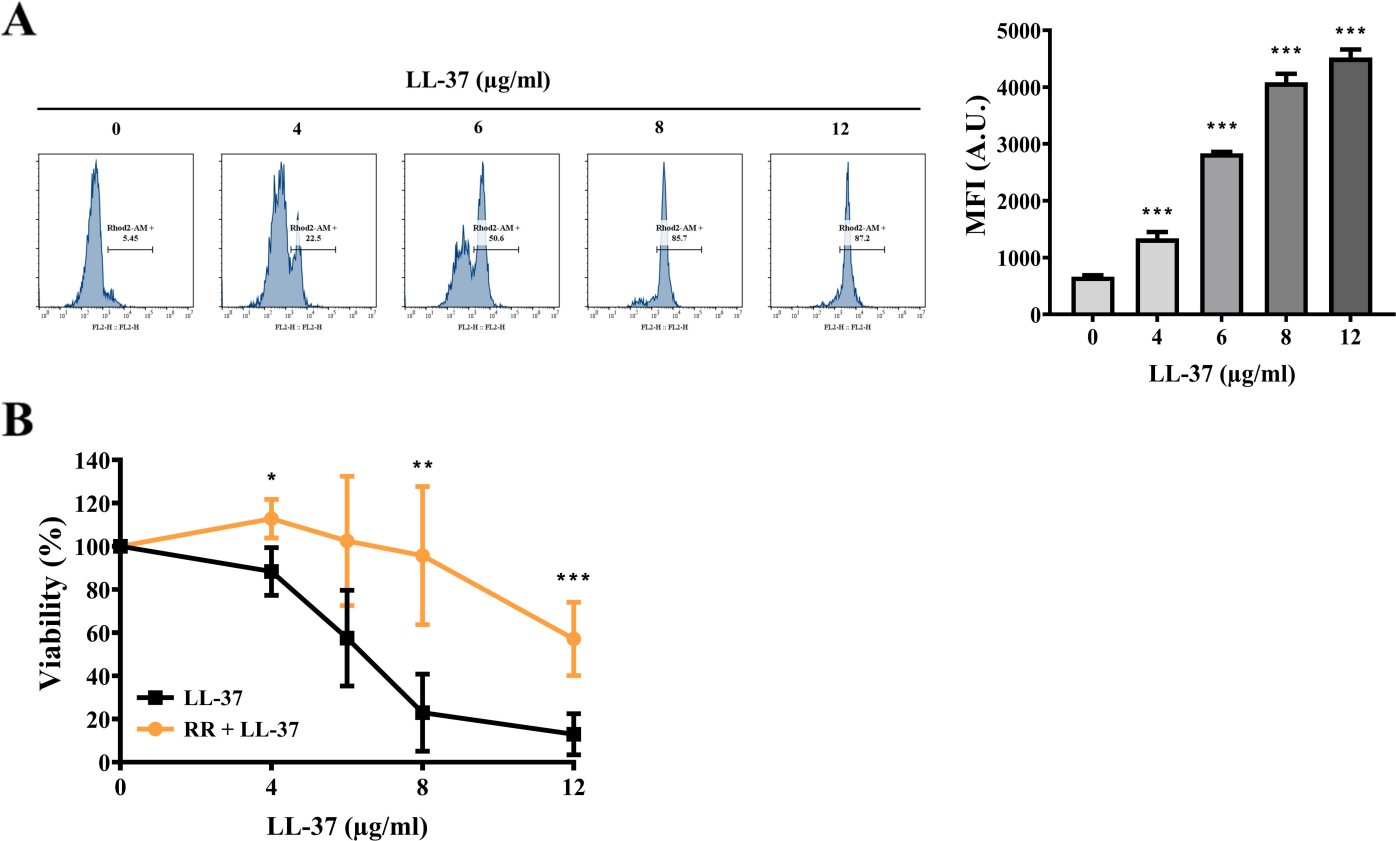
LL-37 induces mitochondrial calcium accumulation. (A) The mitochondrial calcium levels were measured by Rhod-2 AM staining and analyzed using a flow cytometer. The results are expressed as mean ± SD of three independent experiments. ***P < 0.001. A.U.: arbitrary units. (B) The correlation between the killing activity of LL-37 and mitochondrial calcium was also determined. Cells were pretreated with RR, followed by treatment with different concentrations of LL-37 as indicated. Cell viability was calculated and expressed as percentage as compared to the control without RR treatment. All the results are expressed as mean ± SD of three independent experiments. *P < 0.05, **P < 0.01, and ***P < 0.001.

Because LL-37 alters intracellular calcium levels (Fig. 2A and B), we are therefore interested in further examining whether LL-37 impacts mitochondrial calcium levels. To assess the effects of LL-37 on mitochondrial calcium, cells were stained with Rhod-2 AM. The cell-permeable Rhod-2 AM is a fluorescent calcium indicator with a weak positive charge to accumulate in the highly polarized mitochondrial matrix (42). Interestingly, increased levels of mitochondrial calcium were observed in *C. albicans* cells treated with different concentrations of LL-37 compared to the control without treatment (Fig. 3A). To correlate mitochondrial calcium and killing activity of LL-37, ruthenium red (RR) was also used to pre-stain *C. albicans* cells and followed by LL-37 treatment. RR is a known mitochondrial calcium channel inhibitor (43). Our results showed that RR can rescue cells treated with various concentrations of LL-37 (Fig. 3B). In sum, these results suggest that mitochondrial calcium accumulation is relevant to the candidacidal activity of LL-37.

### LL-37 promotes ROS production and causes mitochondrial dysfunction

In mitochondria, changes in calcium homeostasis are associated with ROS generation (44). Elevated levels of mitochondrial calcium stimulate the activity of enzymes involved in cellular respiration, leading to an increase in electron leakage and ROS accumulation (44, 45). Moreover, the overload of mitochondrial calcium can lead to the opening of the permeability transition pore of mitochondria to promote cytochrome C and proton release, resulting in the loss of mitochondrial membrane Δψ, mitochondrial dysfunction, and eventually cell death (44, 45). Because LL-37 enhances both cytosolic and mitochondrial calcium levels, as demonstrated above, ROS production and mitochondrial dysfunction induced by LL-37 were, therefore, further determined.

**Fig 4:**
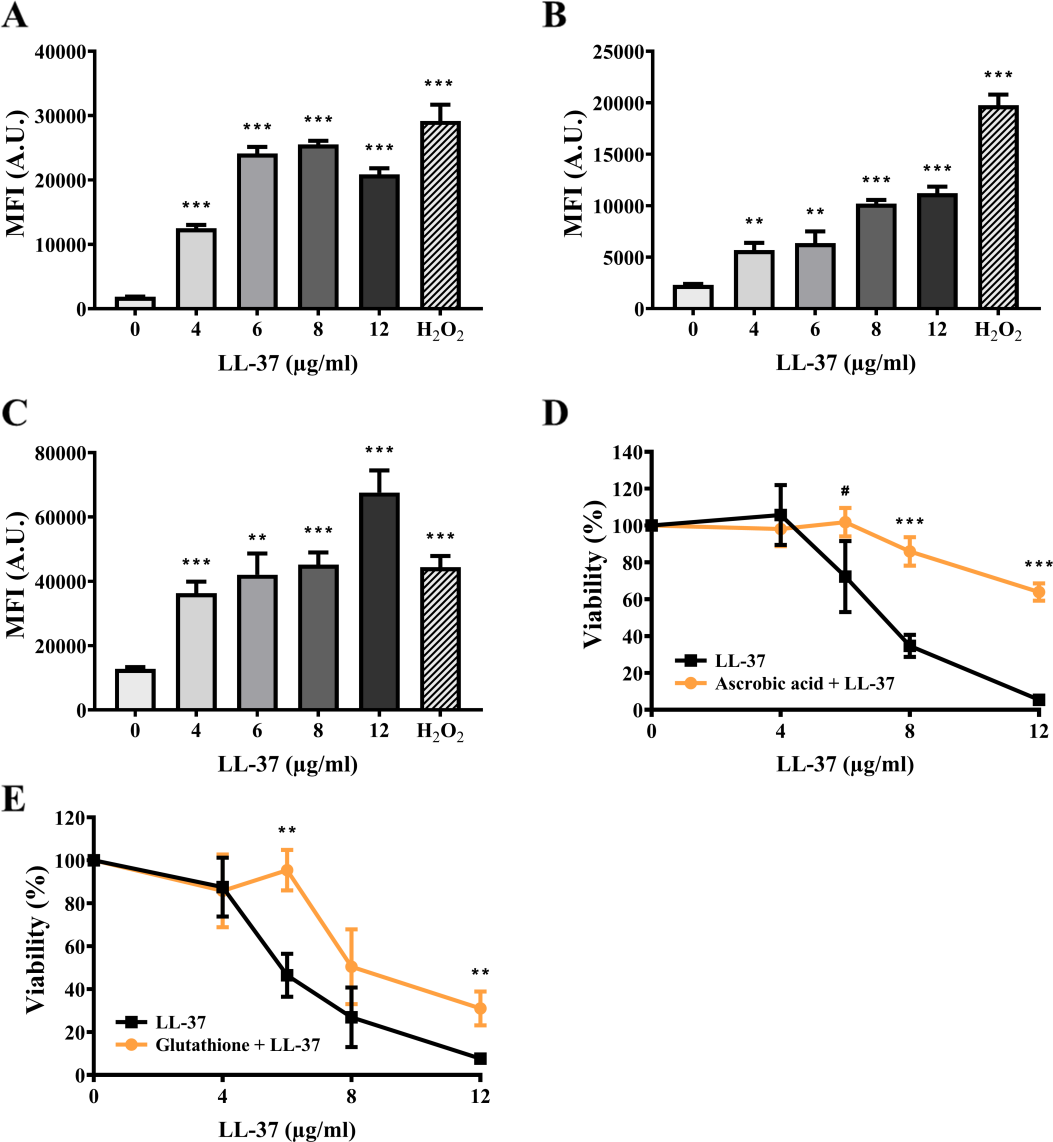
LL-37 promotes cellular and mitochondrial ROS production. Total cellular ROS was measured by (A) DHE and (B) H_2_DCFDA staining and analyzed using a flow cytometer. Cells treated with 15 mM H_2_O_2_ for 1 h were used as the positive control. The results are expressed as mean ± SD of three independent experiments. **P < 0.01 and ***P < 0.001. A.U.: arbitrary units. (C) The mitochondrial ROS level was measured by MitoSOX Red staining and analyzed using a flow cytometer. The results are expressed as mean ± SD of three independent experiments. **P < 0.01 and ***P < 0.001. The effect of ROS scavengers on the killing activity of LL-37 was also determined. Cells were pretreated with (D) ascorbic acid and (E) glutathione, followed by treatment with LL-37. Cell viability was calculated and expressed as percentage with respect to the control without ROS scavenger treatment. The results are presented as mean ± SD of three independent experiments. **P < 0.01, ***P < 0.001, and *#P* = 0.06.

The fluorescent probes DHE (dihydroethidium) and H_2_DCFDA (2′,7′-dichlorodihydrofluorescein diacetate) were used to measure cellular ROS, whereas the MitoSOX Red superoxide indicator was used to detect ROS generated in mitochondria. As shown in Fig. 4A through C, the MFI of all three dyes was increased in cells treated with different concentrations of LL-37 compared to that without LL-37 treatment. These results indicate that LL-37 enhances cellular and mitochondrial ROS production. Moreover, ROS scavenging was performed by ascorbic acid and glutathione to verify the effects of ROS on cell death. Cells were pretreated with the scavengers and then treated with or without LL-37. The results indicated that ascorbic acid and glutathione could rescue cells from LL-37-induced killing (Fig. 4D and E).

**Fig 5:**
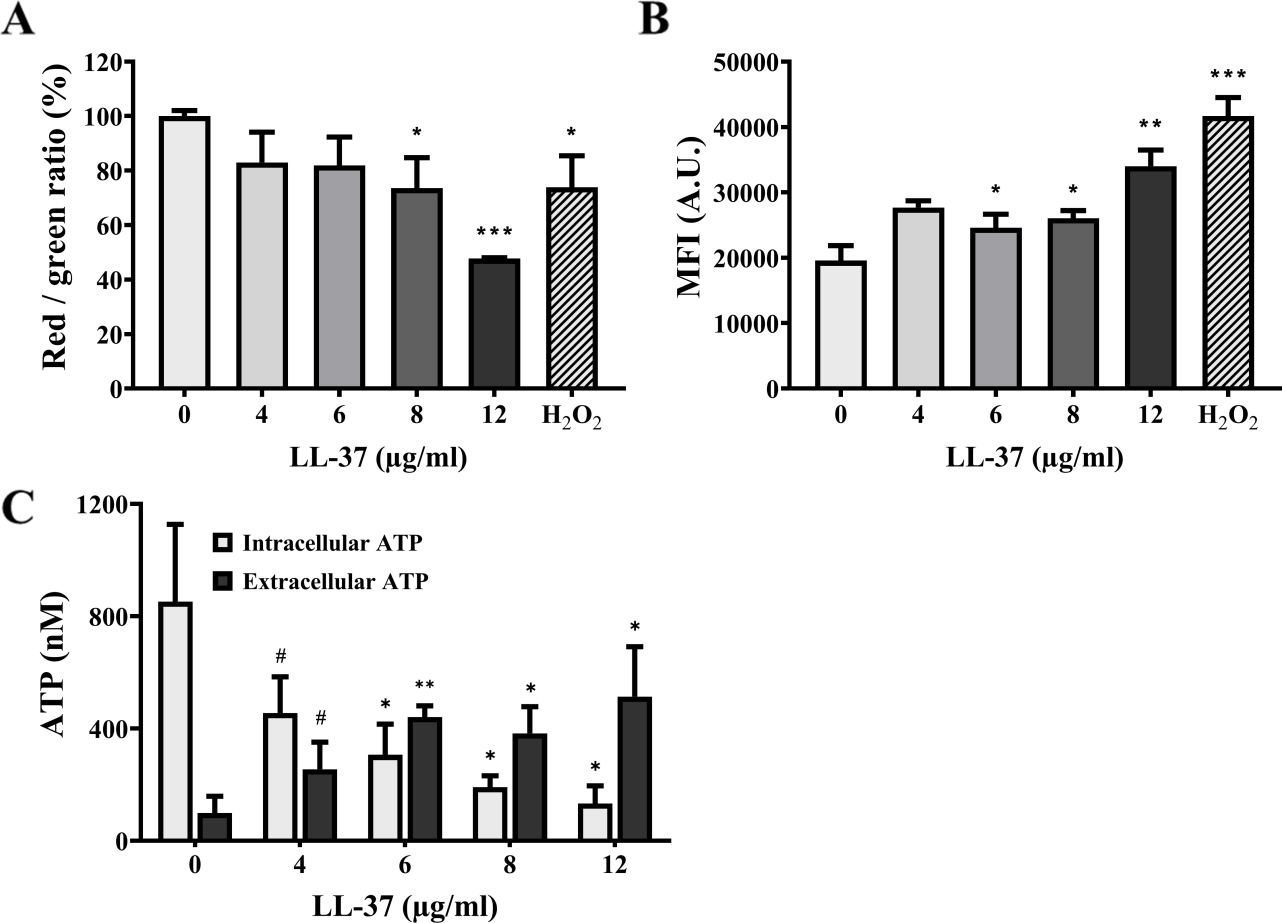
LL-37 causes mitochondrial dysfunction. Mitochondrial membrane potential was measured by (A) JC-1 and (B) Rhodamine 123 staining and flow cytometry. Cells treated with 15 mM H_2_O_2_ for 1 h were used as the positive control. The results are expressed as mean ± SD of three independent experiments. *P < 0.05, **P < 0.01, and ***P < 0.001. A.U.: arbitrary units. (C) The intracellular and extracellular ATP levels of each sample were calculated from an ATP standard curve. The results are presented as mean ± SD of at least three independent experiments. *P < 0.05, **P < 0.01, ***P < 0.001, and #*P* = 0.08.

To examine whether LL-37 can cause mitochondrial dysfunction, mitochondrial Δψ was measured using JC-1 and Rhodamine 123, which selectively accumulate in mitochondria. When the mitochondrial Δψ is high, JC-1 aggregates and shows intense red fluorescence (*λ*_emission_ ~ 590 nm) (46). However, at low membrane potential, JC-1 is present as monomers and exhibits green fluorescence (*λ*_emission_ ~ 530 nm) (46). In Fig. 5A, a decrease in the red/green fluorescence intensity ratio was observed, suggesting mitochondrial depolarization in cells treated with LL-37.

On the other hand, the fluorescent dye Rhodamine 123 specifically stains respiring mitochondria, and mitochondrial energization can induce fluorescence quenching of this dye (46). However, Rhodamine 123 is released into mitochondria when the mitochondrial membrane is depolarized, thus unquenching this dye (46). In Fig. 5B, an increase in the MFI of Rhodamine 123 was revealed in cells treated with LL-37, suggesting mitochondrial membrane depolarization induced by LL-37.

The intracellular ATP level is critical in mitochondria due to its central role in bioenergetics (44). Mitochondria are vital organelles responsible for ATP synthesis through oxidative phosphorylation (44). Thus, any impairment or dysfunction in mitochondria can reduce ATP production (47). Considering the membrane-disturbing activity of LL-37 (Fig. 1A through C), there is a possibility that ATP might leak out of the cells, thereby influencing the measurement of intracellular ATP levels (14). Therefore, the extracellular ATP levels were also determined in addition to assessing the ATP levels within the cells. An increase in extracellular ATP levels and a decrease in intracellular ATP levels were observed, suggesting that LL-37 treatment impacts ATP levels within the cell (Fig. 5C). Together, our results suggest that LL-37 exerts its effects on mitochondria by causing calcium accumulation, ROS generation, and mitochondrial dysfunction.

### LL-37 affects vacuole morphology and properties

In yeast cells, the vacuole is a major calcium storage site that regulates cellular calcium homeostasis (48, 49). In addition, vacuoles change morphology through membrane fusion and fission in response to environmental stresses (50). For example, increased levels of cytosolic calcium correlate with changes in vacuolar morphology and function (50, 51). Finally, the docking stage of yeast vacuolar fusion triggers calcium release into the cytosol, and calcium is required for both vacuolar membrane fusion and fission (52). Interestingly, an interaction among the vacuoles, ROS, and mitochondria has been uncovered in *C. albicans* (45, 53).

**Fig 6:**
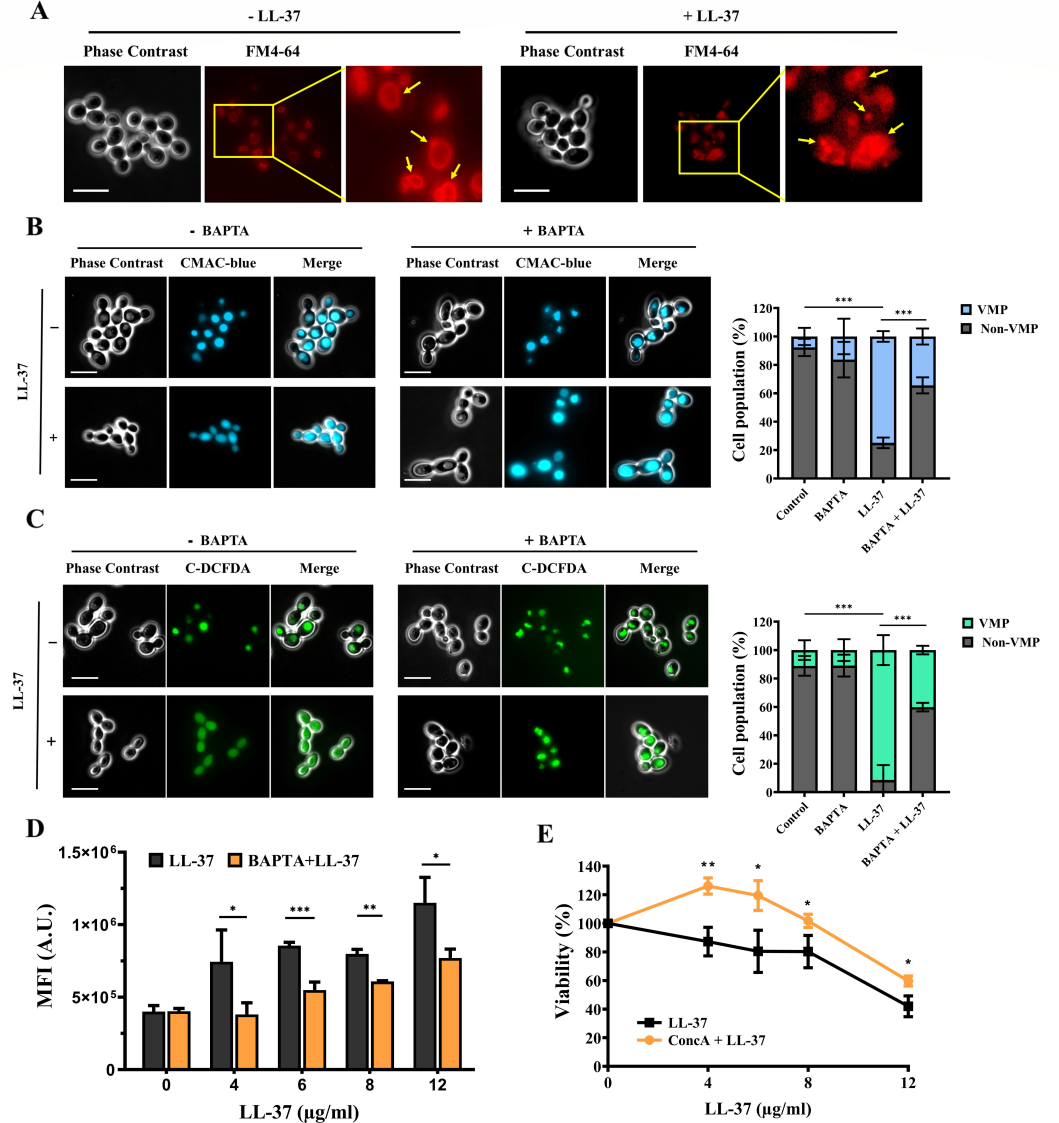
LL-37 affects vacuole morphology and properties. (A) For visualizing vacuole morphology, cells treated with or without LL-37 were stained with the FM4-64 dye and examined by fluorescence microscopy. Yellow arrows point to the fragmented and intact vacuoles in cells treated with and without LL-37, respectively. Scale bar: 10 µm. For the VMP assay, cells were stained with (B) CMAC-blue (7-amino-4-chloromethylcoumarin) and (C) C-DCFDA and examined by fluorescence microscopy. Scale bar: 10 µm. VMP: the vacuole membranes of cells become permeabilized. At least 300 cells in each sample group were examined to determine the VMP and non-VMP cells. The results are presented as mean ± SD of three independent experiments. ***P < 0.001. (D) Vacuole pH was assessed by BCECF-AM [2′,7′-bis-(2-carboxyethyl)-5-(and-6)-carboxyfluorescein, acetoxymethyl ester] staining and analyzed using a flow cytometer. The results are expressed as mean ± SD of three independent experiments. *P < 0.05, **P < 0.01, and ***P < 0.001. A.U.: arbitrary units. (E) To determine the correlation between vacuole acidification and the killing activity of LL-37, cells were pretreated with the V-ATPase inhibitor concanamycin A (ConcA), followed by treatment with LL-37. Cell viability was calculated and expressed as percentage with respect to the control without LL-37 treatment. The results are expressed as mean ± SD of three independent experiments. *P < 0.05, **P < 0.01, and ***P < 0.001.

Because LL-37 affects mitochondria and ROS generation, we are interested in further investigating the impacts of LL-37 on the vacuole. To examine the changes in vacuolar morphology possibly caused by LL-37 treatment, the lipophilic dye FM4-64 was used. This dye selectively stains yeast vacuolar membranes and is commonly used to visualize vacuolar morphology (54). In Fig. 6A, LL-37-treated cells stained with FM4-64 showed a different vacuolar morphology compared to the control without LL-37 treatment. The control cells showed spherical vacuolar morphology, whereas cells treated with LL-37 exhibited a fragmented vacuole segregation structure (Fig. 6A), suggesting LL-37 affects vacuolar morphology.

The permeability and acidification were measured to determine whether LL-37 can also alter the essential properties of the vacuole. For assessment of vacuolar membrane permeability, the membrane-permeable thiol-reactive dyes CMAC-blue (7-amino-4-chloromethylcoumarin) and C-DCFDA [5-(6)-carboxy-2′,7′-dichlorofluorescein diacetate] were used (53, 55). When entering the living cell, CMAC-blue and C-DCFDA can easily enter the vacuole, reacting with vacuolar esterases to become fluorescent and membrane impermeant (56, 57). However, if the vacuolar membrane is compromised, these agents can be released into the cytosol and widely distributed throughout the cells, referred to as vacuolar membrane permeabilization or VMP (53, 55). Coincidentally, as shown in the representative images and the percentage of the cell population (Fig. 6B and C), the fluorescent signal of either CMAC-blue or C-DCFDA was largely evenly distributed within the cytosol in cells treated with LL-37, whereas the fluorescence was mostly observed within the vacuole in the control cells without LL-37 treatment. Moreover, the cell population of VMP was significantly reduced when cells were pretreated with the calcium chelator BAPTA, followed by LL-37 treatment, compared to cells treated with BAPTA or LL-37 alone (Fig. 6B and C). These results indicate that LL-37 promotes VMP, and calcium is somehow associated with the enhanced VMP caused by LL-37.

Appropriate vacuolar acidification plays a key role in maintaining vacuolar functions, and the vacuolar proton pumping ATPase (V-ATPase) controls organelle acidification in eukaryotic cells (58, 59). To measure vacuolar pH, we employed the pH-sensitive dye BCECF-AM [2′,7′-bis-(2-carboxyethyl)-5-(and-6)-carboxyfluorescein, acetoxymethyl ester], which localizes to the vacuole in yeast (60). As shown in Fig. 6D, the MFI of BCECF-AM was increased in cells treated with different concentrations of LL-37 compared to that of the control without LL-37 treatment, suggesting changed vacuolar pH caused by LL-37. Moreover, the MFI of BCECF-AM was reduced when cells were pretreated with BAPTA, followed by LL-37 treatment, compared to cells without LL-37 treatment or treated with BAPTA alone (Fig. 6D). Finally, to correlate vacuolar acidification with the candidacidal activity of LL-37, cells were pretreated with or without the V-ATPase inhibitor concanamycin A (ConcA), followed by incubation with different concentrations of LL-37, and the number of CFUs was counted (61). Figure 6E shows that cells pretreated with ConcA were more resistant to LL-37 than cells without ConcA pretreatment. Therefore, our findings (Fig. 6A through E) suggest a link between vacuolar morphology, properties, and LL-37-induced killing.

### The Rim101 pathway is involved in the killing activity of LL-37

LL-37 affects the PM and also alters calcium homeostasis, as previously demonstrated. These findings raise the possibility that LL-37 may exert its effects by interacting with a PM-coupled receptor to initiate signal transduction and generate cellular responses in *C. albicans*. Therefore, also of interest is to identify the signaling pathway involved in *C. albicans* response to LL-37 treatment.

The Rim pathway consists of an intricate sensor complex in the plasma membrane (Rim8, Rim9, and Rim21/Dfg16), the endosomal sorting complex required for transport proteins, two other Rim proteins (Rim13 and Rim20), and the transcription factor Rim101 (62). In *C. albicans*, the Rim101 pathway is known for controlling pH responses, antifungal tolerance, virulence, and host-pathogen interactions (63 – 65). Intriguingly, this pathway also recognizes lipid asymmetry changes in the PM (66, 67). Moreover, Rim101 appears to mediate calcium fluctuation, control vacuolar acidification, and regulate expression of the ER/Golgi calcium pump gene *PMR1* and the vacuolar calcium ATPase gene *PMC1* (68 – 70). Given the effects of LL-37 on disrupting plasma membrane and calcium homeostasis, we hypothesized that the Rim101 pathway might contribute to the cellular response to LL-37.

**Fig 7:**
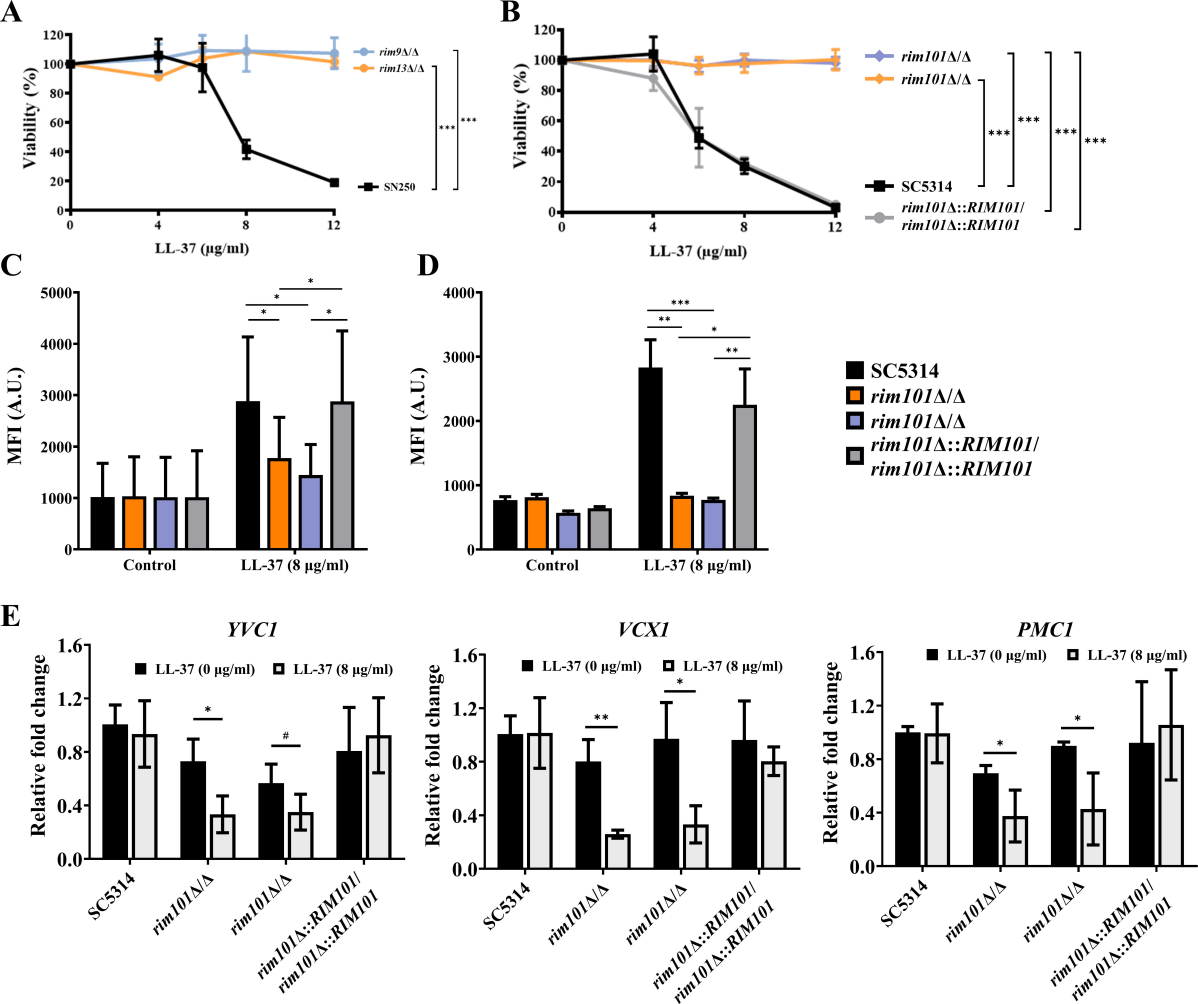
The Rim101 pathway is involved in the cellular response to LL-37. Cell viability of the (A) *rim9*Δ/Δ, *rim13*Δ/Δ, and (B) *rim101*Δ/Δ mutants with LL-37 treatment was assessed. SN250 and SC5314, the parental strains; *rim101*Δ::*RIM101*/*rim101*Δ::*RIM101*, the *RIM101*-reintegrated strain. The results are expressed as mean ± SD of three independent experiments. ***P < 0. 001.The cytosolic mitochondrial calcium levels were measured by (C) Fluo-3 AM and (D) Rhod-2 AM staining and flow cytometry. (E) The expression of genes encoded vacuolar calcium import and export proteins was detected using real-time qPCR. The *ACT1* transcripts were used as an internal control. The results are presented as the mean ± SD of at least three independent experiments. *P < 0.05, **P < 0.01, ***P < 0.001, and #*P* = 0.07.

To test this possibility, the LL-37 susceptibility of three *C. albicans* mutants lacking components of the Rim101 pathway (*rim9*Δ/Δ, *rim13*Δ/Δ, and *rim101*Δ/Δ) was first examined in the buffered LYM medium (see Materials and Methods). The results showed that the parental SN250 or SC5314 strain was sensitive to LL-37, whereas all the mutant strains were resistant to LL-37 (Fig. 7A and B). Moreover, cytosolic and mitochondrial calcium was then assessed using Fluo-3 AM and Rhod-2 AM staining in the wild type, *rim101*Δ/Δ, and *RIM101*-reintegrated strains with and without LL-37 treatment. Interestingly, LL-37 can increase the MFI of both Fluo-3 AM and Rhod-2 AM in the wild type and *RIM101*-reintegrated strains but not in the *rim101*Δ/Δ mutants (Fig. 7C and D). Finally, calcium accumulation inside vacuoles is mainly governed by vacuolar import and export proteins, including the calcium channel Yvc1, the calcium pump Pmc1, and the calcium/H^+^ exchanger Vcx1 (71). Expression of these vacuolar calcium-related genes was detected using reverse transcription (RT) quantitative PCR (qPCR) analysis. In Fig. 7E, we found that the expression of all these vacuolar transporter genes was altered in the *rim101*Δ/Δ mutants upon LL-37 treatment.

In addition, to further correlate the Rim101 pathway with membrane disruption by LL-37, the PM Δψ, PM permeability, and lipid droplet accumulation were assessed in the *rim101*Δ/Δ mutants with and without LL-37 treatment. In Fig. S2, the percentages of DiBAC4(3)- and Sytox Green-positive cells and the MFI of Nile red were all increased in the wild type and *RIM101*-reintegrated cells treated with LL-37 compared to that without treatment. Interestingly, the intensities of all three dyes had no significant differences in the *rim101*Δ/Δ mutants treated with and without LL-37. However, it is of note that the *rim101*Δ/Δ mutants showed a higher percentage of Sytox Green-positive cells compared to the wild type and *RIM101*-reintegrated strains without LL-37 treatment. Although the detailed mechanism needs to be further studied, one possible explanation is that Rim101 also plays a role in the maintenance of PM lipid asymmetry, a vital determinant of PM properties (67). These results suggest a connection between the Rim101 pathway and alterations in PM properties caused by LL-37.

Collectively, our results support the notion that the Rim101 pathway is involved in cellular responses to LL-37 on changing membrane properties, calcium homeostasis, and *C. albicans* killing.

## DISCUSSION

LL-37 is a multifunctional AMP of the human cathelicidin family with broad-spectrum antimicrobial activities. Previous studies found that LL-37 mainly associates with the cell surface of *C. albicans* by interacting with cell wall components, including polysaccharides and the exoglucanase Xog1 (16 – 18). LL-37 treatment alters cell wall composition and architecture, affects cell wall remodeling, and induces cell wall β-1,3-glucan exposure (19). Ultimately, LL-37 causes *C. albicans* cell aggregation and reduces cell adhesion to abiotic surfaces *in vitro* and epithelial surfaces *in vivo* (16).

In this study, in addition to its known interaction with the cell wall, we further demonstrated that LL-37 also acts on the PM of *C. albicans*. LL-37 treatment induced PM depolarization, permeability, and membrane stress-associated lipid droplet accumulation (Fig. 1A through C). Consistently, an early study indicated that PM permeabilization is correlated with the inhibition of *C. albicans* cell growth by LL-37 (19, 72). However, cell wall and membrane disruption may not be the only mechanism by which LL-37 kills *C. albicans*. As demonstrated by genome-wide expression profiling, LL-37 modulates the expression of genes with various functions, including transporters, regulators for biological processes, and stress response (19). These results suggest multiple mechanisms of LL-37 against *C. albicans*. Hence, to expand our current understanding regarding LL-37 targeting cell surface integrity, intracellular responses of *C. albicans* to LL-37 were studied herein.

Calcium ions are involved in many cellular processes, and calcium homeostasis is crucial for the survival and pathogenicity of *C. albicans* (35). Nevertheless, excessively high intracellular calcium accumulation can trigger cell death (73). Importantly, mitochondria play a key role in regulating intracellular calcium by uptake and buffering cytosolic calcium (73). In many diverse eukaryotes, calcium uptake is mediated by the mitochondrial calcium uniporter (MCU) complex and is driven by the membrane potential across the mitochondrial inner membrane (74, 75). Although lacking MCU, electrochemical gradients across the inner mitochondrial membrane are sufficient to drive mitochondria calcium uptake in yeast (76, 77). In the present study, we detected an increase in both cytosolic and mitochondrial calcium levels in LL-37-treated cells, and the elevated levels of calcium were related to the killing activity of LL-37 (Fig. 2 and 3). However, because the components and mechanisms involved in mitochondrial calcium influx and efflux have not been characterized in *C. albicans*, the details of the impacts of LL-37 on cellular and mitochondrial calcium homeostasis still need to be unraveled.

In mammalian cells, the affinity of the MCU for calcium is very low (*K*_*D*_ of 20–30 µM) under physiological conditions (75). Moreover, the cytosolic calcium concentration is generally insufficient to activate the MCU for mitochondrial calcium uptake (75). However, this puzzle is partially solved by the findings of the interaction between mitochondria and the ER, the largest calcium storage site in mammalian cells. The mitochondria-ER interaction creates high calcium microdomains to provide adequate calcium concentrations needed for activating MCU (78). While MCU is lacking, the mutual interplay between different organelles also occurs in yeast cells (79). For the interaction between ER and mitochondria, the ER-mitochondria encounter structure (ERMES) has been identified in *Saccharomyces cerevisiae* and *C. albicans* (80). The ERMES complex is involved in organelle tethering and transfer of calcium ions and lipids (81).

To date, the study of the association among ER, mitochondria, and calcium homeostasis has attracted much attention (45, 81). For example, mitochondrial dysfunction promotes the loss of redox balance and activates ER-derived ROS generation in *S. cerevisiae* (82). Moreover, ROS production by mitochondria and rapidly decreased calcium levels in the ER lumen are common features of ER stress and activation of the unfolded protein response (UPR) (45, 83). Finally, intracellular calcium overloading can enhance mitochondrial calcium uptake, causing changes in many mitochondrial properties, including membrane potential (45).

Our previous study found that LL-37 activates UPR, induces ER-derived ROS, and affects ER-associated protein secretion (20). Nevertheless, we also determined the effects of LL-37 on the mitochondria in this study. In Fig. 3B, the relationship between *C. albicans* killing by LL-37 and mitochondrial calcium accumulation was evaluated by cells pretreatment with and without RR, an inhibitor blocking mitochondrial calcium channel. Although RR is known for inhibiting mitochondrial calcium uptake in yeast, it also impacts ER calcium release possibly by interacting with the inositol 1,4,5-trisphosphate receptor (IP_3_R) on the ER in mammalian cells (84). Therefore, we cannot ignore the possible contribution of the ER to the elevated mitochondrial calcium in cells upon LL-37 treatment (Fig. 3A). Apart from inducing mitochondrial calcium levels, LL-37 promotes mitochondrial ROS generation, alters membrane potential, and causes mitochondrial dysfunction (Fig. 3 to 5). Together, our studies suggest the link between ER and mitochondria is involved in *C. albicans* response to LL-37. However, how LL-37 affects the ER-mitochondria interface and local calcium dynamic at this interference requires further study.

Additionally, mitochondria also interact with vacuoles through novel membrane contact sites, called the vacuole and mitochondria patch or vCLAMP in yeast (85). The vCLAMP complex is co-regulated with ERMES and also serves as a backup for ERMES in maintaining mitochondrial and vacuolar functions (86). Notably, unlike ER in mammalian cells, the vacuole is the primary cellular calcium store in yeast (48). In *C. albicans*, oxidative stress and ER stress are found to provoke vacuolar calcium release (87, 88). The release of vacuolar calcium is related to the fusion and fission of the vacuolar membrane and occurs during the docking stage of membrane fusion (50, 52, 89). Besides, the vacuolar Ca^2+^/H^+^ antiporter Vcx1 was previously found to elevate cytosolic calcium and block vacuole acidification important for many cellular processes (89). As demonstrated in Fig. 6A through E *, C. albicans* cells treated with LL-37 exhibited a change in vacuole morphology, an increase in vacuole membrane permeability, and an impairment of vacuole acidification. These findings suggest that LL-37 also exerts its effects on vacuoles, possibly by affecting calcium release from vacuoles into the cytosol. However, the possibility that LL-37 may also affect vacuole calcium uptake and the vacuole-mitochondria interaction cannot be excluded.

Of special interest in this study is the involvement of the Rim101 pathway in the cellular response to LL-37. In this study, deletion mutants of several Rim101 pathway components enhanced cell resistance to LL-37 and attenuated the increase of cytosolic and mitochondrial calcium induced by LL-37 (Fig. 7A through D). In addition, LL-37 also modulated the expression of vacuolar calcium transporter genes in the *rim101*Δ/Δ mutants (Fig. 7E). Lastly, unlike the wild type and *RIM101*-reintegrated strains, there were no significant differences in PM properties in the *rim101*Δ/Δ mutants with and without LL-37 treatment (Fig. S2 ). These results suggest that *C. albicans* response to LL-37 is Rim101-dependent. However, the mechanisms by which LL-37 activates the Rim101 pathway remain an outstanding question. Favorably, previous studies have provided some implications for addressing this question. First, the Rim101 pathway is activated in response to altered lipid asymmetry of the PM and possibly recognizes the surface charges of *S. cerevisiae* (67, 90). Secondly, the Rim101 pathway is triggered by detecting distinct stresses via the Rim21-dependent sensing complex (67, 90). The Rim21 complex senses stress conditions such as extracellular alkalization and altered lipid asymmetry in the PM, followed by activating the Rim101 cascade to regulate transcriptional response to stress (67). Finally, the PM can physically interact with the ER to form ER–PM contact sites, which are the interfaces for signaling, lipid synthesis and exchange, and ion transport (91, 92). Interestingly, the Rim101 pathway also participates in ER stress response by sensing the status of the PM and could be constitutively activated by ER–PM contact site disruption (91). Therefore, to better understand how the Rim101 pathway is activated by LL-37, the action of LL-37 on the PM should be further investigated. Specifically, whether LL-37 induces lipid asymmetry alteration at the PM and whether the sensor complex of the Rim101 pathway and the ER–PM contact sites involved in LL-37 sensing are still unknown. These issues are currently under investigation in our laboratory.

**Fig 8:**
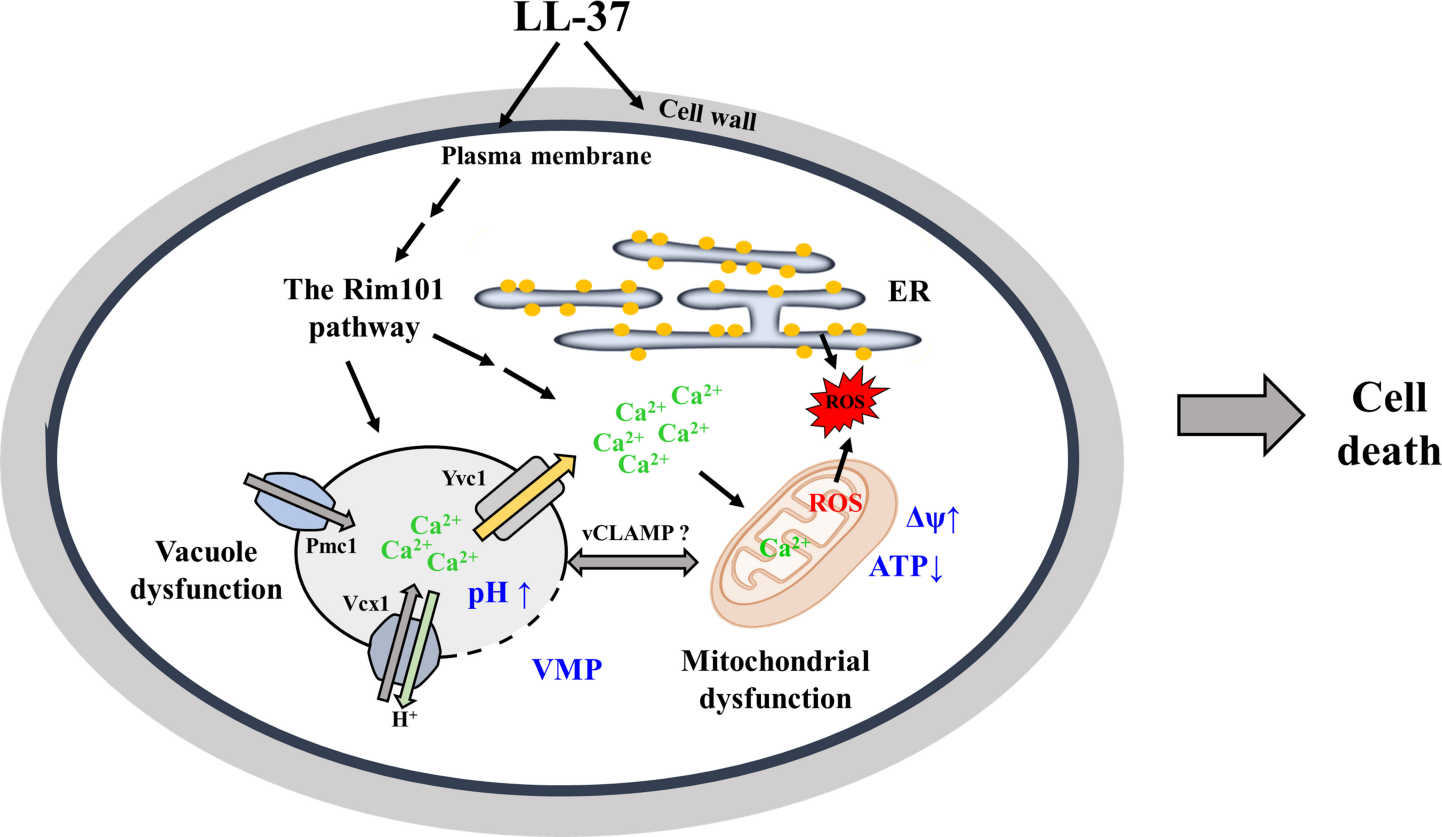
Mechanisms of action of LL-37 against *C. albicans*. Based on previous studies and the present work, LL-37 is mainly associated with cell surface of *C. albicans*. However, it has profound impacts on multiple cellular components and biological processes. LL-37 induces cell surface perturbation, demonstrated by cell wall remodeling, β-1,3-glucan exposure, and cell adhesion reduction. Moreover, LL-37 also causes alteration in plasma membrane potential and permeability. The cell surface perturbation may thus affect the sensor complex that triggers the Rim101 pathway. Consequently, cellular and organelle calcium homeostasis is altered. The resulting calcium imbalance is accompanied by excessive generation of ROS, depolarization of mitochondrial membrane, and reduction of mitochondrial ATP synthesis. Additionally, LL-37 treatment also leads to change in vacuole morphology, increases in vacuole pH, and enhancement of VMP. The changes in vacuolar features are associated with calcium accumulation in vacuoles. Finally, we previously demonstrated that LL-37 induces ER stress and ER-derived ROS production. Together, fungicidal action against *C. albicans* by LL-37 is complex, involving functions of multiple organelles, maintenance of calcium homeostasis, responses to ROS, and cellular signaling. However, whether protein complexes of organelle communications, e.g., vCLAMP (for vacuole and mitochondria interaction) and ERMES (for ER and mitochondria interaction), are affected by LL-37 still needs to be investigated. Δψ: membrane potential.

In conclusion, as outlined in Fig. 8, we have demonstrated that LL-37 exerts its effects against *C. albicans* with multiple modes of action. Moreover, cellular response to LL-37 is complex and involves the interplay among various organelles, calcium homeostasis, ROS, and signaling. Finally, our results suggest the potential use of LL-37 and other AMPs in antifungal therapy.

## MATERIALS AND METHODS

### Peptide and reagents

LL-37 (LLGDFFRKSKEKIGKEFKRIVQRIKDFLRNLVPRTES) was synthesized by MDBio, Inc. (Taipei, Taiwan). The peptide was >95% pure, determined by reverse-phase high-performance liquid chromatography. Molecular weight of the peptide was verified using mass spectrometry. The stock solution of LL-37 (5 mg/mL) was prepared by dissolving the peptide in sterile double-distilled water (ddH_2_O) and stored at −20°C before use. All reagents were purchased from Sigma-Aldrich (St. Louis, MO, USA) unless stated otherwise.

### *C*. *albicans* strains and growth conditions

*C. albicans* strains used in this work are listed in Table S1 (in the supplemental material). Cells were routinely stored at −80°C and plated on yeast extract-peptone-dextrose (YPD) agar plates (1% yeast extract, 2% peptone, 2% glucose, and 1.5% agar) before each experiment. A single colony was inoculated into YPD broth and grown overnight (~16 h) at 30°C with shaking (180 rpm). Cells were then subcultured in 5 mL YPD broth (with an initial concentration of ~1.5 × 10^7^ CFU/mL) and grown at 30°C to the exponential phase for further experiments.

### Strain construction

The *rim101*Δ/Δ and *RIM101*-reintegrated strains were generated using the *SAT1*-flipper method, and the procedures for strain construction are described in the supplemental material. In addition, the primers used are listed in Table S2.

### Cell susceptibility to LL-37 and FK506

Cell susceptibility to LL-37 and/or the calcineurin inhibitor FK506 was determined by colony-forming unit (CFU) counting. In brief, *C. albicans* cells were harvested by centrifugation, washed twice with sterile ddH_2_O, and resuspended in LYM medium (adjusted to a pH value of 6.0 using 10 mM potassium phosphate buffer) (93). Subsequently, cells (~1.2 × 10^7^ CFU/mL) were treated with or without different concentrations of LL-37 and/or FK506 (16 µg/mL) and incubated with shaking (180 rpm) at 37°C, 5% CO_2_ for 1 h. Cells were then diluted with sterile phosphate-buffered saline (PBS), grown on YPD agar plates at 30°C, and CFUs were counted.

### Measurement of plasma membrane potential and permeability

*C. albicans* plasma membrane potential and permeability were measured by DiBAC4(3) (Invitrogen, Carlsbad, CA, USA) and Sytox Green (Invitrogen, Carlsbad, CA, USA) staining, respectively. Briefly, after treatment with or without different concentrations of LL-37, *C. albicans* cells were harvested, washed twice with PBS, resuspended in PBS containing either 20 µg/mL DiBAC4(3) or 25 nM Sytox Green, and incubated for 30 min at 30°C in the dark. Cells were measured using an Accuri C6 flow cytometer (BD Biosciences, San Jose, CA, USA), and data were analyzed using the BD FlowJo software (version 10.8.1).

### Measurement of lipid droplet accumulation

Nile red staining was performed to determine the accumulation of lipid droplets caused by LL-37 (31). Cells were treated with different concentrations of LL-37, washed twice with PBS, and resuspended in 5 µg/mL Nile red solution. After incubation at 30°C for 30 min, cells were centrifuged, washed three times with PBS, and the fluorescence intensity was measured using an Accuri C6 flow cytometer (BD Biosciences).

### Measurement of cytosolic and mitochondrial calcium concentration

Cytosolic calcium concentration was measured using two calcium-sensitive dyes, Fluo-3 AM and Fura-2 AM. For the measurement of mitochondrial calcium, a positively charged calcium-binding dye Rhod-2 AM was used. *C. albicans* cells were treated with or without different concentrations of LL-37, harvested by centrifugation, and washed twice with Krebs buffer (pH 7.2) composed of 132 mM NaCl, 4 mM KCl, 1.4 mM MgCl_2_, 6 mM glucose, 10 mM HEPES, 10 mM NaHCO_3_, and 1 mM CaCl_2_ (94). The cells were then mixed with 5 µM Fluo-3 AM, 5 µM Fura-2 AM, or 10 µM Rhod-2 AM supplemented with 1% bovine serum albumin and 0.01% Pluronic F-127 (in Krebs buffer) and incubated at 28°C for 30 min. After staining, the cells were washed twice with calcium-free Krebs buffer at 37°C for 30 min. Finally, cells were washed three times with PBS and resuspended in PBS. The fluorescence intensities of Fluo-3 AM (*λ*_emission_ = 526 nm) and Rhod-2 AM (*λ*_emission_ = 581 nm) were measured using an Accuri C6 flow cytometer (BD Biosciences). The fluorescence intensity of Fura-2 AM (*λ*_excitation_ = 340 nm, *λ*_emission_ = 505 nm) was measured using a Victor Nivo multimode microplate reader (PerkinElmer).

Moreover, to correlate mitochondrial calcium and *C. albicans* killing by LL-37, cells were pretreated with or without 0.5 mM RR at 30°C for 30 min, followed by treatment with or without LL-37. Then, the number of CFUs was counted as described above.

### Measurement of cellular and mitochondrial ROS

Cellular ROS accumulation was measured by two fluorescent dyes DHE and H_2_DCFDA (95). Moreover, MitoSOX Red (Invitrogen, Carlsbad, CA, USA) was used to detect mitochondrial ROS (95). *C. albicans* cells were treated with or without different concentrations of LL-37, collected, and washed twice with PBS. Then, cells were mixed with 20 µM DHE, 20 µg/mL H_2_DCFDA, or 5 µM MitoSOX Red and incubated at 30°C for 10–20 min in the dark. The fluorescence intensities were measured using an Accuri C6 flow cytometer (BD Biosciences).

### Calcium chelation and cell rescue assay

To determine the relationship between calcium and *C. albicans* killing by LL-37, calcium chelators were used in cell rescue assay. After growing overnight, *C. albicans* cells were subcultured in YPD with and without BAPTA (final concentration 0.6 mM), EDTA (final concentration 0.6 mM), or BAPTA-AM (final concentration 50 µM) and grown at 30°C for approximately 3 h. Cells with calcium chelation were then washed twice with sterile ddH_2_O, resuspended in LYM medium, and mixed with or without different concentrations of LL-37. After incubation at 37°C for 1 h, CFUs were then counted as described above.

To link ROS generation and *C. albicans* killing by LL-37, ROS scavengers were used to determine whether cells treated with LL-37 could be rescued. Cells were pretreated with 100 mM ascorbic acid and 50 µM glutathione at 30°C for 30 min. After pretreatment, cells were harvested, washed, resuspended in LYM, and treated with or without different concentrations of LL-37 for 1 h, and CFUs were then counted.

### Assessment of mitochondrial membrane potential

The mitochondrial membrane Δψ was measured by JC-1 and Rhodamine 123 staining, as previously described (46, 95), with some modifications. *C. albicans* cells were treated with or without different concentrations of LL-37 for 1 h. After centrifugation, cells were harvested, washed twice with PBS, resuspended in 25 µM Rhodamine 123, and incubated for 10 min at 30°C in the dark. For JC-1 staining, cells treated with or without LL-37 were harvested, mixed with 2 µM JC-1, and incubated for 15 min at 37°C in the dark. After staining, cells were washed three times with PBS, and the fluorescence intensity was subsequently measured using an Accuri C6 flow cytometer (BD Biosciences). Cells without LL-37 treatment were used as negative controls, whereas cells treated with 15 mM hydrogen peroxide (H_2_O_2_) were used as positive controls.

### Measurement of intracellular and extracellular ATP levels

Cells (~1.2 × 10^7^ CFU/mL) were incubated with or without different concentrations of LL-37 with shaking (180 rpm) at 37°C, 5% CO_2_ for 1 h, and harvested by centrifugation. The supernatant was placed on ice for extracellular ATP measurement, while the cell pellets were broken as previously described to measure intracellular ATP levels (95).

For ATP measurement, an ATP Determination Kit (A22066, Invitrogen) was used according to the manufacturer’s instructions. Briefly, a reaction buffer composed of 0.5 mM D-luciferin, 1.25 µg/mL firefly luciferase, and 1 mM DTT was freshly prepared before each experiment. Subsequently, 10 µL of the supernatant or cell lysate was mixed with 90 µL of the reaction buffer. The mixture was then transferred to a black 96-well microplate, and luminescence was detected at 560 nm using a Victor Nivo multimode microplate reader (PerkinElmer). A standard curve of different concentrations (0, 10, 100, and 1,000 nM) of ATP was generated. The ATP concentration of each sample was then calculated from the standard curve.

### Investigating the effects of LL-37 on the vacuole

Morphology, permeability, and acidification of the vacuole were examined to determine the effects of LL-37 on the vacuole. For examining vacuole morphology, cells were stained with 20  µM FM4-64 at 30°C for 30  min, harvested by centrifugation, and washed twice with fresh YPD medium. Then, cells were further incubated in YPD at 30°C for 90  min, followed by treatment with or without LL-37 for 1 h (54, 96). Vacuole morphology was examined using a fluorescence microscope (Zeiss Axio Imager A1; Carl Zeiss, Jena, Germany). The images were processed using the ZEISS ZEN software (version 3.3.89.0000).

To assess the occurrence of VMP and its association with calcium homeostasis, cells were pretreated with and without BAPTA (final concentration of 0.6 mM) and grown in YPD at 30°C for ~3 h. Cells were then harvested by centrifugation, followed by incubation with different concentrations of LL-37 at 37°C for 1 h. After treatment, cells were washed twice with PBS and resuspended in 1 µg/mL CMAC-blue or 5 µg/mL C-DCFDA (53, 55). The mixture was incubated at 30°C for 30 min in the dark. After staining, cells were washed three times with PBS and examined using a fluorescence microscope (Zeiss Axio Imager A1; Carl Zeiss, Jena, Germany). To identify a subpopulation of VMP or non-VMP cells, at least 300 cells treated with or without LL-37 were photographed, and the images were used for cell counting.

For the measurement of vacuolar pH, BCECF-AM was used (60). Briefly, cells were pretreated with and without BAPTA (final concentration of 0.6 mM) at 30°C for ~3 h, followed by treatment with different concentrations of LL-37 for 1 h. Cells were then harvested by centrifugation, washed twice with PBS, and resuspended in 5 µM BCECF-AM. The mixture was incubated at 30°C for 30 min in the dark. After staining, cells were washed three times with PBS. The samples were resuspended in PBS, and the fluorescence intensity of BCECF-AM was measured using an Accuri C6 flow cytometer (BD Biosciences).

To correlate the vacuole pH with the killing activity of LL-37, cells were pretreated with 3 µM ConcA at 30°C for 30 min. After pretreatment, cells were harvested, washed, resuspended in LYM, treated with or without different concentrations of LL-37 for 1 h, and CFUs were counted.

### RNA extraction and RT real-time qPCR

Total RNA extraction, RT for cDNA synthesis, and real-time qPCR were performed as previously described (20). The primers used in this study are listed in Table S2. The *ACT1* transcripts were used as an endogenous control for qPCR. All experiments were performed independently and repeated at least three times, and the average CT values were obtained. The relative fold change in the expression of each gene was calculated using the 2^−ΔΔCT^ method (20).

### Statistical analysis

The two-tailed Student’s *t*-test was used to determine significant differences between samples. Statistical significance was indicated with a *P* value <0.05.

## SUPPLEMENTAL MATERIAL

Supplemental material spectrum.02551-23-s0001.pdf Supplemental methods, Fig. S1 and S2, and Tables S1 and S2.

ASM does not own the copyrights to Supplemental Material that may be linked to, or accessed through, an article. The authors have granted ASM a non-exclusive, world-wide license to publish the Supplemental Material files. Please contact the corresponding author directly for reuse.
