# Supplementary material for: Antimicrobial peptide LL-37 disrupts plasma membrane and calcium homeostasis in Candida albicans via the Rim101 pathway
Source: Microbiol Spectr. 2023 Oct 27;11(6):e02551-23. doi: 10.1128/spectrum.02551-23 (PMC10715129; doi:10.1128/spectrum.02551-23)
Supplement: Supplemental material — Supplemental methods, Fig. S1 and S2, and Tables S1 and S2. [file spectrum.02551-23-s0001.pdf]

Supplemental Materials

Strain construction. The *rim101*-deleted (*rim101*Δ/*rim101*Δ) and the *RIM101*-reintegrated (*rim101*Δ::*RIM101*/*rim101*Δ::*RIM101*) strains were generated using the *SAT1*-flipper method (1). The primers used are listed in Table S2. The 5' and 3' flanking regions of *RIM101* were amplified from the SC5314 genome by PCR amplification using the primer pairs RIM101-UR-F-Kpn1 and RIM101-UR-R-XhoI, and RIM101-DR-F-SacII and RIM101-DR-R-SacI, respectively. Then, the amplified 5' and 3' flanking regions of *RIM101* were cloned separately into the pSFS2A vector by restriction enzyme digestion and ligation to generate pSFS2AdRIM101 (1). The DNA fragment carrying the 5' and 3' flanking regions of *RIM101* and the *SAT1*-flipper cassette was excised from pSFS2AdRIM101 via *Kpn1*/*Sac*I digestion. After purification, the linear DNA was transformed and integrated into the *C. albicans* chromosome between the 5' and 3' flanking sequences of *RIM101* via homologous recombination. The transformants were selected by nourseothricin for PCR validation (1). The successful transformants cells were grown in YPM to induce the expression of recombinase controlled by *MAL2* promoter for *SAT1*-flipper excision from the *RIM101* locus. The second allele of *RIM101* was knocked out by the same strategy using the heterozygous *rim101*-deleted mutants (*rim101*Δ/*RIM101*), and the primer pairs RIM101-UR2-F-Kpn1 and RIM101-UR2-R-XhoI, and RIM101-DR-F-SacII and RIM10-DR-R-SacI, respectively.

To generate the *RIM101*-reintegrated strain, the DNA fragment comprised the *RIM101* promoter region, and the full-length *RIM101* coding sequence was amplified using the primer pair RIM101-UR-F-Kpn1 and RIM101-DR-R-SacI from the SC5314 genome. The fragment was cloned into pSFS2AdRIM101 upstream of the *SAT1*-flipper cassette via *Kpn1*/*XhoI* digestion and ligation to replace the original *Kpn1*- *XhoI* fragment, generating pRIM101R. The DNA fragment carrying the full-length *RIM101* coding sequence, the *SAT1*-flipper cassette, and the 5' and 3' flanking regions of *RIM101* were excised from pRIM101R via *Kpn1*/*Sac*I digestion, purified, and transformed into the homozygous *rim101*-deleted strains. The *SAT1*-flipper cassette of the successful transformants selected by nourseothricin was then popped out as previously described (1). Integration of the second allele of *RIM101* was done by the same strategy using the strains carrying the integration in the first allele of *RIM101*. The gene constructions were verified by PCR amplification of *RIM101* locus with the primer pair RIM101-F and RIM101-R (Fig. S1 in the supplemental materials).

1. Reuss O, Vik A, Kolter R, Morschhäuser J. 2004. The SAT1 flipper, an optimized tool for gene disruption in *Candida albicans*. Gene. 341:119-27.

Supplementary Figures

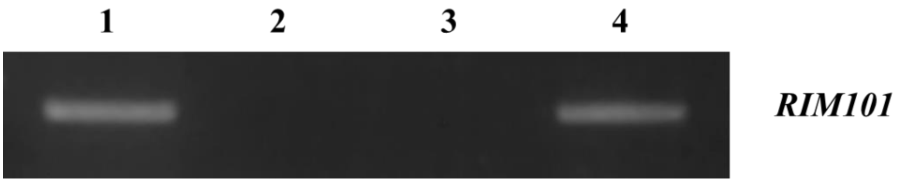

Fig S1. Validation of *RIM101* gene deletion and reintegration by PCR analysis of genomic DNA. 1: wild type; 2, 3: the *rim101*Δ/Δ mutants; 4: the *RIM101*-reintegrated strain.

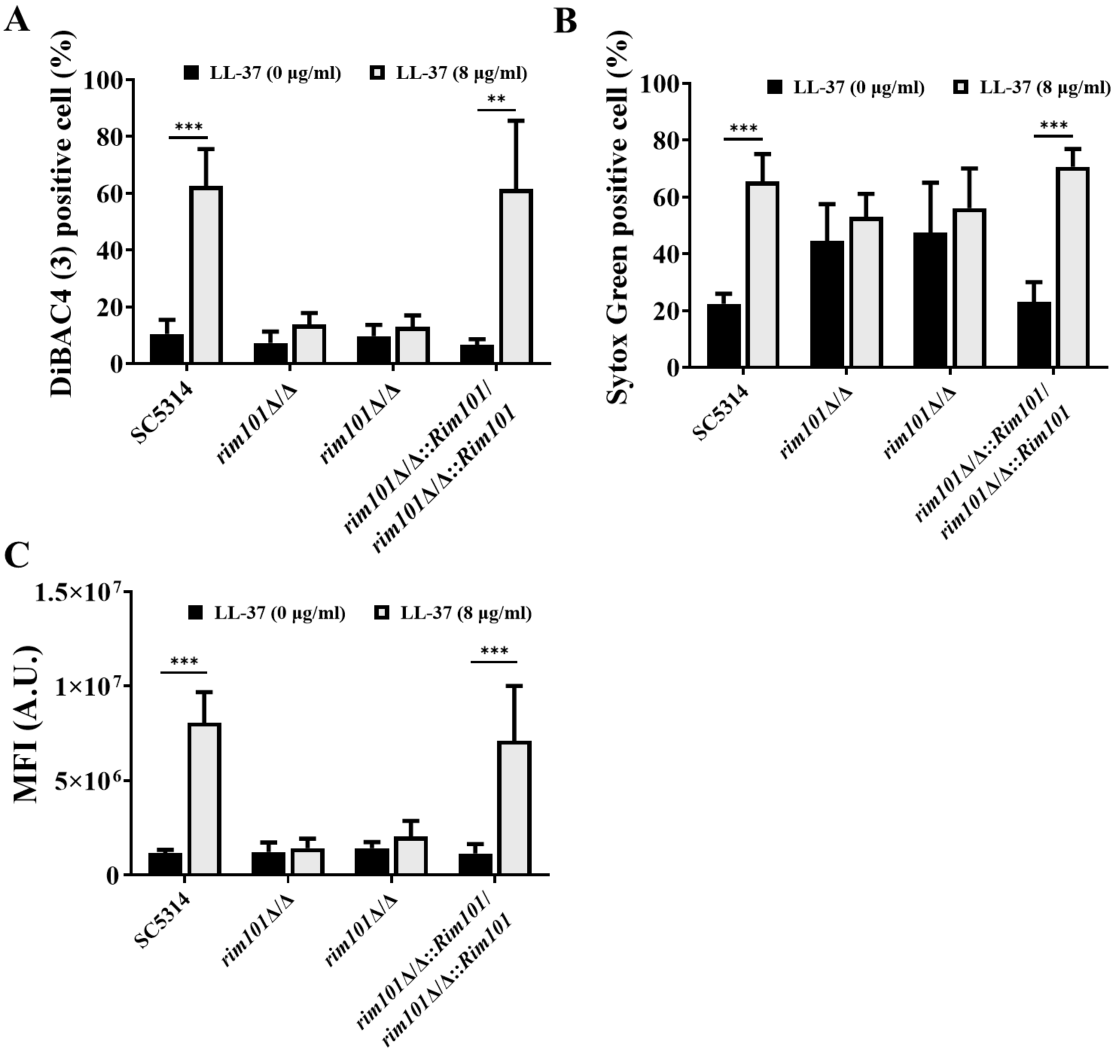

Fig S2. PM properties and lipid droplet accumulation were not affected by LL-37 in the *rim101*Δ/Δ mutants. The PM Δψ and permeability was measured by (A) DiBAC4(3) and (B) Sytox green staining, respectively, and analyzed using a flow cytometer. Lipid droplet accumulation was measured by (C) Nile red staining and analyzed using a flow cytometer. MFI, mean fluorescence intensity; A.U., arbitrary units. The results are presented as mean ± SD of three independent experiments. *** $p < 0.001$.

Supplementary Tables

Table S1. ***C. albicans*** **strains used in this study**

Strain name	Genotype	Source
SC5314	Wild type	[1]
The *rim101*Δ/Δ mutant	*rim101*Δ::*FRT*/*rim101*Δ::*FRT*	This study
RIM101-reintegrated strain	*rim101*Δ:: *RIM101-FRT*/ *rim101*Δ:: *RIM101-FRT*	This study
SN250	*ura3*Δ-*iro1*Δ::*imm*^{434}/*URA3-IRO1, his1*Δ/*his1*Δ, *arg4*Δ/*arg4*Δ, *leu2*Δ::*C.m.LEU2*/*leu2*Δ::*C.d.HIS1*	[2]
The *rim9*Δ/Δ mutant	*rim9*Δ/Δ in SN250	[2]
The *rim13*Δ/Δ mutant	*rim13*Δ/Δ in SN250	[2]

1 Gillum AM, Tsay EY, Kirsch DR. Isolation of the *Candida albicans* gene for orotidine-5'- phosphate decarboxylase by complementation of *S. cerevisiae* ura3 and E. coli pyrF mutations. Mol Gen Genet. 1984;198(2):179-182.
2 Noble SM, French S, Kohn LA, Chen V, Johnson AD. 2010. Systematic screens of a *Candida albicans* homozygous deletion library decouple morphogenetic switching and pathogenicity. Nat Genet. 42(7):590-8.

Table S2. Primers used in this study

Primer	Sequences (5' to 3')
For strain construction[a]	
RIM101-DR-F-SacII	GATCCGCGGGAAGAAGTGACGATTGATGG
RIM101-DR-R-Sac I	TCAGAGCTCATAGAGAACGGTAACACTACTTTC
RIM101-UR-F-Kpn1	AAGGTACCTAATGGTTGTTTA AGCTTCGC
RIM101-UR-R-XhoI	CTCACTCGAGTGTCTAAAAATCTCGTTTGTCTAG
RIM101-UR2-F-Kpn1	AAGGTACCAAGTAGAGCCGAGAAATGAC
RIM101-UR2-R-XhoI	CTCACTCGAGATTGAACAACATCACACTACC
RIM101-F	TGGCGGTAACATCTATGAAG
RIM101-R	GCAGATTTCTGGTAGGTTGA
For real-time qPCR	
q-ACT1-F	ATACTCTGTCTGGATTGGTGGTTCT
q-ACT1-R	TTTTGAAATCCACATTTGTTGGA
q-YVC1-F	ACCGATTCAAGGGAGTTCCA
q-YVC1-R	CAAGTCCCACTCGGTGTCAA
q-VCX1-F	GCTGCTTTCCATGCCTCATTA
q-VCX1-R	CCCCGGTTCAGGAAAACC
q-PMC1-F	GGGAGTTGCTGAAGCCGTATT
q-PMC1-R	CCCGGTGACCATACGAACA

[a]The restriction sites are underlined.
